# The impact of Cysteine-Rich Intestinal Protein 1 (CRIP1) in human breast cancer

**DOI:** 10.1186/1476-4598-12-28

**Published:** 2013-04-09

**Authors:** Natalie Ludyga, Sonja Englert, Kerstin Pflieger, Sandra Rauser, Herbert Braselmann, Axel Walch, Gert Auer, Heinz Höfler, Michaela Aubele

**Affiliations:** 1 Institute of Pathology, Helmholtz Zentrum München, German Research Center for Environmental Health, Ingolstaedter Landstrasse 1, Neuherberg, 85764, Germany; 2 Department of Radiation Cytogenetics, Helmholtz Zentrum München, German Research Center for Environmental Health, Ingolstaedter Landstrasse 1, Neuherberg, 85764, Germany; 3 Department of Oncology and Pathology, Karolinska Institute and Hospital, Stockholm, S-17176, Sweden; 4 Institute of Pathology, Technische Universität München, Trogerstrasse 18, Munich, 81675, Germany

**Keywords:** CRIP1, HER2, ERB-B2, Breast cancer, Tumorigenesis, Prognosis, Invasion, RNAi, RNA interference, Proliferation

## Abstract

**Background:**

CRIP1 (cysteine-rich intestinal protein 1) has been found in several tumor types, its prognostic impact and its role in cellular processes, particularly in breast cancer, are still unclear.

**Methods:**

To elucidate the prognostic impact of CRIP1, we analyzed tissues from 113 primary invasive ductal breast carcinomas using immunohistochemistry. For the functional characterization of CRIP1, its endogenous expression was transiently downregulated in T47D and BT474 breast cancer cells and the effects analyzed by immunoblotting, WST-1 proliferation assay and invasion assay.

**Results:**

We found a significant correlation between CRIP1 and HER2 (human epidermal growth factor receptor 2) expression levels (*p* = 0.016) in tumor tissues. In Kaplan Meier analyses, CRIP1 expression was significantly associated with the distant metastases-free survival of patients, revealing a better prognosis for high CRIP1 expression (*p* = 0.039). Moreover, in multivariate survival analyses, the expression of CRIP1 was an independent negative prognostic factor, along with the positive prognosticators nodal status and tumor size (*p* = 0.029). CRIP1 knockdown in the T47D and BT474 breast cancer cell lines led to the increased phosphorylation of MAPK and Akt, to the reduced phosphorylation of cdc2, and to a significantly elevated cell proliferation *in vitro* (*p* < 0.001). These results indicate that reduced CRIP1 levels may increase cell proliferation and activate cell growth. In addition, CRIP1 knockdown increased cell invasion *in vitro*.

**Conclusions:**

Because the lack of CRIP1 expression in breast cancer tissue is significantly associated with a worse prognosis for patients and low endogenous CRIP1 levels *in vitro* increased the malignant potential of breast cancer cells, we hypothesize that CRIP1 may act as a tumor suppressor in proliferation and invasion processes. Therefore, CRIP1 may be an independent prognostic marker with significant predictive power for use in breast cancer therapy.

## Background

Breast cancer is the most common cancer diagnosed among women in the Western world and is the leading cause of female cancer death [1]. The determination of the hormone receptor status (estrogen (ER) and progesterone (PR)) has become standard practice in the management of invasive breast cancers and is useful as a prognostic and predictive factor [2]. Similarly, human epithelial growth factor receptor 2 (HER2) positivity, which is observed in approximately 30% of breast cancers, is an important marker for selecting targeted therapy with the monoclonal anti-HER2 antibody trastuzumab (Herceptin™) [2-6]. Because a portion of HER2-overexpressing tumors is nonresponsive to Herceptin™ therapy, there is a need to identify additional markers linked to HER receptors and associated signaling proteins for the development of other targeted therapeutic treatments.

CRIP1 (cysteine-rich intestinal protein 1) belongs to the LIM/double-zinc finger protein family and has been shown to be overexpressed in several tumor types, including breast, cervical, prostate, pancreatic, and colorectal cancers [7-11]. However, little is known regarding its prognostic impact and functional role in human cancers. Previous studies have revealed an association between CRIP1 and HER2 levels in breast cancer cells. In breast cancer cell lines and human breast cancer tissues, an overexpression of HER2 was correlated with an overexpression of CRIP1 [4,12,13]. A recent study on an intestinal type of gastric cancer reported that the overexpression of CRIP1 was an independent predictor of shortened survival [14]. Patients with a high expression of CRIP1 displayed decreased survival probabilities compared with patients with low expression levels of CRIP1. Conversely, in osteosarcomas, CRIP1 expression was more frequently found in patients with long-term survival and without metastases, indicating a favorable prognostic effect [15].

To date, there is no functional characterization of CRIP1, and its precise role in cancer cells and its impact in prognosis are still unclear. The aim of this study was to analyze the prognostic impact and functional role of CRIP1 in human breast cancer. Using FFPE tissues from invasive ductal breast carcinomas (IDC), we show an association between CRIP1 expression and histopathological parameters and, the clinical course of the disease. Additionally, we identified functional properties of CRIP1 in two permanent breast cancer cell lines using RNA interference (RNAi).

## Results

### Association between CRIP1 and immunohistochemical and histopathological parameters

**Figure 1:**
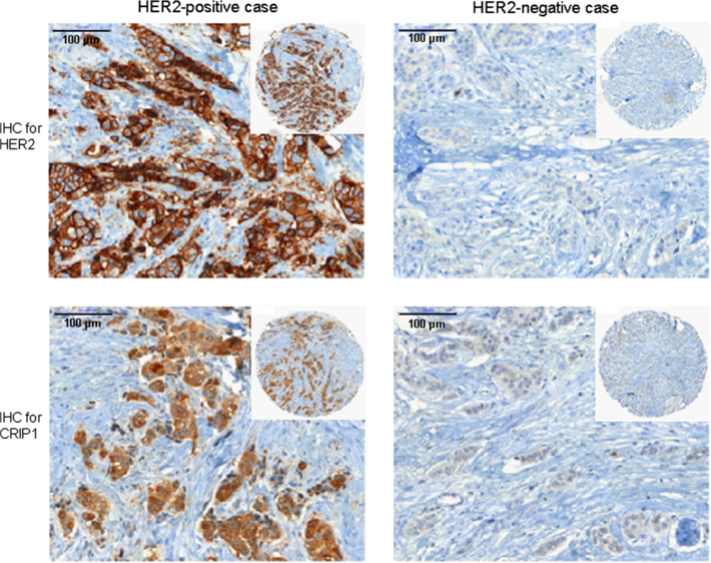
**The co-expression of HER2 and CRIP1 in breast cancer tissue.** Representative images of breast cancer tissues showing positive or negative immunohistochemical staining for HER2 and CRIP1, respectively.

We found no or low CRIP1 expression in 79 tumors (37 were negative and 42 were classified as 1+), medium expression in 20 tumors (classified as 2+) and high CRIP1 expression in 14 tumors (classified as 3+). In breast cancer tissue, positive and negative staining of CRIP1 was frequently associated with HER2 staining (Figure 1). A significant correlation was found between CRIP1 and the expression of HER2 (*p* = 0.016), and an inverse correlation was found between CRIP1 expression and estrogen receptor (ER, *p* = 0.04). No significant association was identified between CRIP1 and lymph node status, tumor size, histological grade, or progesterone receptor expression.

### Impact of CRIP1 on the clinical course of patients

**Figure 2:**
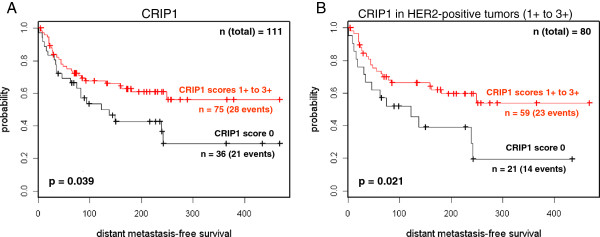
**Kaplan Meier survival analysis of the distant metastases-free survival of patients.** (**A**) Patients were grouped according to CRIP1 expression into negative (0) and positive (classified 1+ to 3+) groups. (**B**) Only patients with HER2-positive tumors were stratified according to their CRIP1 expression (negative vs. positive).

In univariate analyses of the distant metastases-free survival of the patients, a significant positive correlation was found between CRIP1 expression (*p* = 0.039) and a more favorable prognosis for patients with positive CRIP1 expression (classified 1+ to 3+) (Figure 2A, Table 1). HER2 expression was not significantly associated with the clinical course of the disease (*p* = 0.8). In the tumor cohort analyzed, there was no significant association between CRIP1 expression and the lymph node status of the patients. However, when we considered only lymph node-positive tumors, a trend was observed between CRIP1 positivity and a better clinical course of the disease (*p* = 0.09) (Table 1).

**Table 1:** Results from univariate and multivariate survival analyses for a distant metastases-free survival of breast cancer patients

**CRIP1**				
	negative (0)	36	21 / 58%	
	positive (1+, 2+, 3+)	75	28 / 37%	**p = 0.039**
**CRIP1 in lymph node-negative tumors**				
	CRIP1 negative (0)	23	11 / 48%	
	CRIP1 positive (1+, 2+, 3+)	44	11 / 25%	p = 0.1 n.s.
**CRIP1 in lymph node-positive tumors**				
	CRIP1 negative (0)	13	10 / 77%	
	CRIP1 positive (1+, 2+, 3+)	33	17 / 52%	p = 0.09 n.s.
**CRIP1 in HER2-negative tumors** (0)				
	CRIP1 negative (0)	14	7 / 50%	
	CRIP1 positive (1+, 2+, 3+)	14	5 / 36%	p = 0.6 n.s.
**CRIP1 in HER2-positive tumors** (1+, 2+, 3+)				
	CRIP1 negative (0)	21	14 / 67%	
	CRIP1 positive (1+, 2+, 3+)	59	23 / 39%	**p = 0.021**
**Multivariate Cox regression analysis**				
	coefficient	chi^2^	p-value	
nodal status	0.86	7.45	0.006	
tumor size	0.51	4.58	0.03	
CRIP1	−0.66	4.28	0.039 *	total **p = 0.029**

*inverse correlation, Significant p-values are in bold.

The CRIP1 expression in our tumor cohort was associated with the expression of HER2 (*p* = 0.016). Considering the CRIP1 expression in only HER2-negative tumors (Table 1), no significant association was found with the clinical course. However, in HER2-positive tumors, two different prognostic groups could be identified according to the CRIP1 expression. CRIP1-positive tumors showed a better prognosis, with 39% of patients (23 out of 59) suffering distant metastases compared with 67% (14 of 21) of CRIP1-negative patients, within the follow-up period of more than 30 years (*p* = 0.021) (Figure 2B, Table 1). This result clearly indicates that CRIP1 expression may be a useful prognostic marker in HER2-positive tumors.

Remarkably, in multivariate Cox regression analysis, CRIP1 proved to be an independent prognostic factor, along with nodal status (pN) and tumor size (pT) (*p* = 0.039) (Table 1).

### Co-expression of HER2 and CRIP1 in the T47D and BT474 breast cancer cell lines

**Figure 3:**
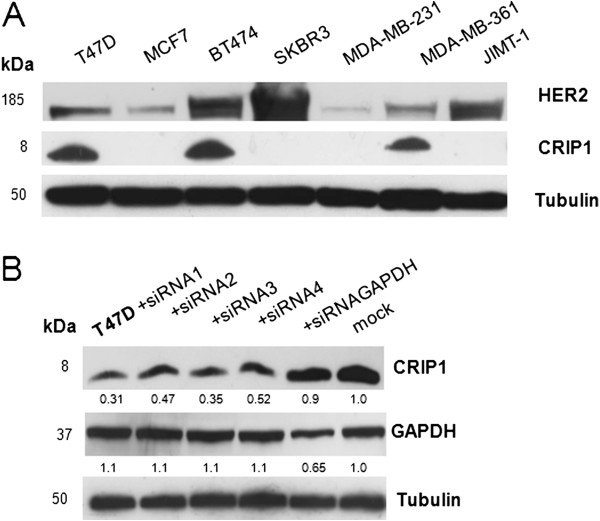
**CRIP1 protein levels in breast cancer cell lines and after transient downregulation in T47D cells.** (**A**) Western blot analysis of HER2 and CRIP1 expression in seven breast cancer cell lines using antibodies targeting HER2, CRIP1 and tubulin (loading control). (**B**) Western blot analysis of CRIP1 expression in nontransfected T47D cells (mock) and transiently transfected T47D cells using four different siRNAs and siRNAGAPDH (positive control).

For functional *in vitro* analyses, appropriate breast cancer cell lines were identified that co-expressed both HER2 and CRIP1 at adequate levels. The adequate co-expression of both proteins was detected in the T47D, BT474 and MDA-MB-361 cell lines (out of seven analyzed breast cancer cell lines) (Figure 3A). In this study, we selected T47D and BT474 cells for CRIP1 knockdown and subsequent analyses because in these cells the protein expression levels of CRIP1 and HER2 were higher than in the MDA-MB-361 cells.

### The downregulation of CRIP1 significantly elevates the cell proliferation *in vitro*

**Figure 4:**
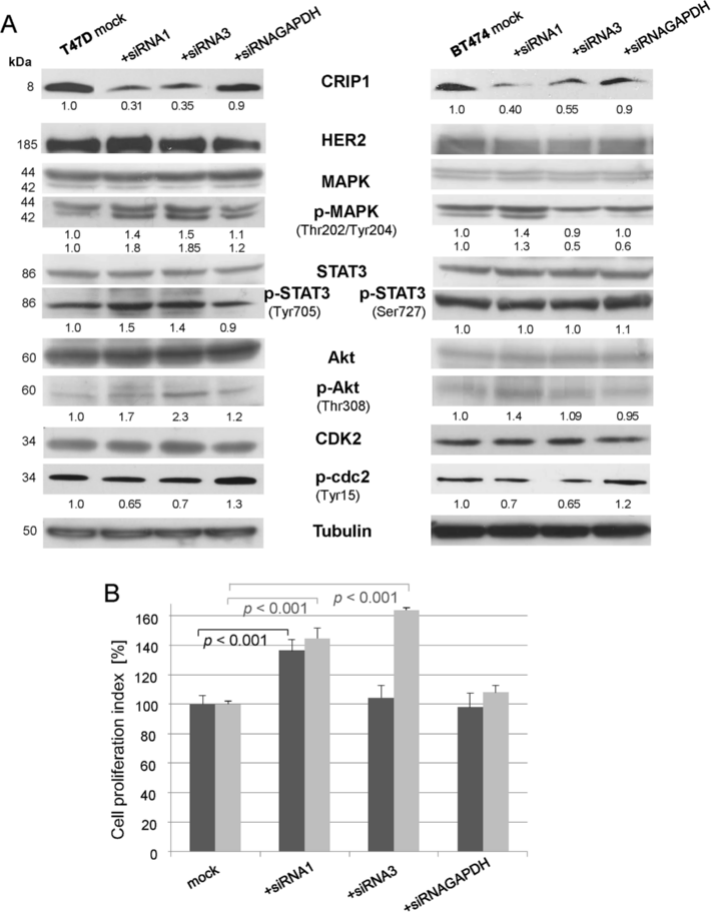
**CRIP1 silencing results in the activation of signaling proteins involved in cell proliferation.** (**A**) Western blot analysis showing the expression and phosphorylation levels of signaling proteins after the knockdown of CRIP1 in T47D and BT474 breast cancer cells using HER2, (phospho) MAPK, (phospho) STAT3, (phospho) Akt and (phospho) cdc2 antibodies. Tubulin was used as a loading control. The mean values of three independent experiments are shown. (**B**) Seventy-two hours after transfection, the WST-1 reagent was added to a defined amount of T47D and BT474 cells and the absorbance measured after 3 h is shown. The graph represents the amount of viable cells in relation to the mock control. The means of five independent experiments, the standard deviations and the *p*-values are shown. For statistical analyses, the student´s t-test was performed.

After the identification of small interfering RNAs (siRNAs) that showed specific and efficient CRIP1 downregulation (Figure 3B and Additional file 1), the effects of CRIP1 knockdown in the T47D and BT474 cells on the expression and phosphorylation of HER2 signaling-associated proteins were analyzed using immunoblotting. Following CRIP1 knockdown, no effects were observed for HER2 (human epidermal growth factor receptor 2), and HER2-related and proliferation-associated signaling proteins like MAPK (mitogen-activated protein kinase), STAT3 (signal transducer and activator of transcription 3), Akt, cdk2 (cyclin dependent kinase 2) or PTEN (phosphatase and tensin homologue deleted on chromosome ten) protein expression levels (Figure 4A). In contrast, the phosphorylation of MAPK at Thr202/Tyr204 was increased in both cell lines due to CRIP1 downregulation (Figure 4A). This mitogen-activated protein kinase is involved in cell proliferation, differentiation and growth [16]. Phosphorylated MAPK activates the downstream phosphorylation of its substrates in the cytoplasm, or it translocates to the nucleus and subsequently regulates gene expression through the phosphorylation of transcription factors. p38 MAPK regulates cell survival and apoptosis [16]. The phosphorylation of p38 MAPK at Thr180/Tyr182 leads to the activation of other MAPK and transcription factors that also regulate apoptosis. CRIP1 knockdown did not lead to an altered phosphorylation of p38 MAPK (data not shown). Akt activation leading to the regulation of survival and apoptosis is regulated by phosphorylation at Thr308 and Ser473 [17]. CRIP1 knockdown led to an increased phosphorylation of Akt at Thr308 (Figure 4A).

The phosphatase PTEN is a tumor suppressor that negatively regulates the PI3K/Akt pathway [18]. The phosphorylation of PTEN impairs its tumor suppressive function. CRIP1 silencing did not affect the phosphorylation of PTEN (data not shown).

STAT3 drives cell growth, survival, differentiation and gene expression via phosphorylation at Tyr705. The phosphorylation at Ser727 is associated with its role as a transcription factor [19]. After the siRNA-mediated downregulation of CRIP1, we did not observe an altered phosphorylation of STAT3 at Ser727, but the phosphorylation at Tyr705 was elevated in T47D cells (Figure 4A), in BT474 cells this phosphorylation site was not detectable. We further analyzed the expression and phosphorylation of cell cycle proteins in response to changes in CRIP1 expression. No altered expression was observed for cyclin E, cyclin D1, cyclin A proteins or the cyclin-dependent kinase 2 (data not shown). However, we observed a reduced phosphorylation of cdc2 at Tyr15 in both cell lines following CRIP1 silencing (Figure 4A).

In addition, we investigated the proliferation of T47D and BT474 cells following CRIP1 knockdown based on the enzymatic cleavage of tetrazolium salts into formazan (WST-1 proliferation assay). Compared with control cells (mock and cells transfected with siRNA targeting GAPDH) the proliferation was significantly elevated of approximately 40% when T47D cells were depleted of CRIP1 using the most efficient siRNA1 (Figure 4B). In BT474 cells, in both silencing approaches the proliferation index was elevated of over 40% or 60%, respectively (Figure 4B).

### CRIP1 silencing enhances the invasion of breast cancer cells

**Figure 5:**
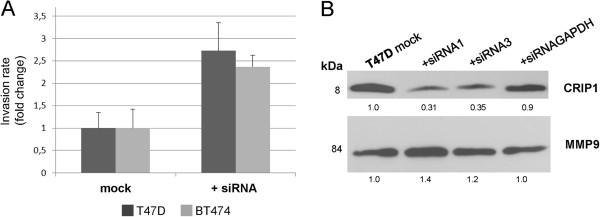
**CRIP1 knockdown increases the invasive potential of T47D and BT474 breast cancer cells *in vitro*.** (**A**) A quantification of the invasion assay of nontransfected T47D cells or BT474 cells (mock) compared with transiently transfected T47D cells or BT474 cells 48 h post-transfection (T47D cells) and 72 h post-transfection (BT474 cells). The mean values and standard deviations of two independent experiments are shown. (**B**) Western blot analysis showing the expression of cleaved (84 kDa band) MMP9 in supernatants of nontransfected T47D cells (mock) and T47D cells with transiently downregulated CRIP1 expression using effective siRNAs and siRNAGAPDH (positive control). The mean values of three independent experiments are shown.

To further elucidate the functional role of CRIP1 in breast cancer, we analyzed the migration or invasion of transfected and control T47D and BT474 breast cancer cells. Due to a non-confluent cell formation, the BT474 cells are not suitable for a wound scratch assay. The migration of T47D cells was not affected by reduced CRIP1 protein levels (data not shown). In contrast, compared with control cells, the invasion of T47D cells was 2.7 fold higher after knockdown of the CRIP1 protein using the most efficient siRNA1 (Figure 5A). In addition, the invasion of BT474 cells was also elevated (approximately 2.3 fold higher) following CRIP1 knockdown. To further confirm this observation, we determined the activation of MMP9 (matrix metalloproteinase 9) with the immunoblotting of the supernatants of serum-starved cells. The activation of MMP9 (illustrated by the band at 84 kDa) was slightly increased following CRIP1 silencing in T47D cells (Figure 5B). In the BT474 cell line, the MMP9 protein was not detectable.

## Discussion

CRIP1 was first identified in the mouse small intestine through its pattern of developmental regulation during the neonatal period [20]. It is a member of the LIM/double-zinc finger protein family and is a developmentally regulated protein that appears to play a role in protein-protein interactions during transcriptional processes [21-23]. Members of the LIM zinc-finger protein family are thought to play a role in the growth and differentiation of eukaryotic cells [24,25]. CRIP1 has also been suggested to play a role in the host defense system, and the differential expression of CRIP1 can alter cytokine patterns and the immune response in transgenic mice [24]. The overexpression of CRIP1 has been observed in several human malignant tumors, including cervical cancer, breast cancer, prostate cancer, colorectal cancer, pancreatic cancer, gastric cancer and osteosarcoma [7-11,13-15]. However, no agreement has been reached regarding the results obtained from the tumors of different entities, and the functional role of CRIP1 is still unclear.

In breast cancer, a role for CRIP1 was proposed in HER2-related oncogenesis because the upregulation of CRIP1 was recorded in HER2-overexpressing carcinomas of the breast [4], which indicates an indirect prognostic effect of CRIP1. Furthermore, Rauser et al. confirmed these results using mass spectrometry by identifying CRIP1 expression in HER2-positive breast tumors [13]. In our study on primary breast carcinomas, CRIP1 expression that was detected by IHC was not significantly correlated with HER2 expression. However, regarding the distant metastases-free survival of patients, we demonstrated a more favorable clinical course for HER2-positive tumors that expressed CRIP1 compared with HER2-positive tumors lacking CRIP1.

To the best of our knowledge, a positive association between CRIP1 and the distant metastases-free survival of breast cancer patients has not been described previously. Here, we show that patients with CRIP1-expressing tumors have a more favorable prognosis compared with patients with CRIP1-negative tumors. Moreover, we show that CRIP1 expression in breast carcinomas is of independent (inverse) prognostic value in multivariate survival analyses in addition to lymph node status and tumor size. Baumhoer et al. also found a favorable clinical course for patients with CRIP1 expression in osteosarcoma [15], which fully corresponds to our results in breast carcinomas. However, the inverse prognostic relevance of CRIP1 expression that we identified in our tumor cohort is not in agreement with results obtained in gastric cancers [14]. Studies in gastric cancers have demonstrated that CRIP1 expression is directly associated with a worse prognosis for patients.

CRIP1 was also described in breast cancer to be among a panel of genes relevant to bone metastases [26,27]. In our study, we did not analyze metastases, only primary breast tumors, in which CRIP1 expression was not significantly associated with lymph node metastases or tumor size. Our *in vitro* analyses confirm the findings in metastatic tissues. The invasive behavior of the cells was strongly elevated following CRIP1 knockdown in T47D and BT474 cells. Additionally, we confirmed that the potential for the enhanced invasion of the cells after CRIP1 knockdown may also be based on the increase in active MMP (matrix metalloproteinases) 9 levels. MMPs are key proteins in wound healing, tumor invasion, angiogenesis and carcinogenesis [28]. A prerequisite for invasion and thus tumor malignancy is the cleavage of the precursor protein into the active MMP [29], which, in our study, was elevated after CRIP1 downregulation.

Latonen et al. found that CRIP1 protein expression was upregulated as a response to increased cellular density, indicating a proliferation-reducing activity of CRIP1 [30]. This observation is in agreement with our *in vitro* analyses, suggesting that low CRIP1 protein levels promote cell proliferation.

To further characterize the function of CRIP1 in breast cancer, particularly its role in cell signaling and proliferation processes, we investigated the phosphorylation status of several signaling molecules (MAPK, STAT3, PTEN and Akt). These proteins are all essential in cellular processes, including proliferation, survival, growth, migration, differentiation and anti-apoptotic pathways [16,19,31-33]. Following CRIP1 knockdown, we observed an elevated phosphorylation of MAPK. This kinase promotes proliferation, growth and migration through the phosphorylation of other key regulators and transcription factors. Elevated levels of phosphorylated MAPK due to CRIP1 knockdown could increase the proliferation and growth of breast cancer cells; however the degree of the effects were dependent on the respective cell line and used siRNA. This outcome may correlate with different genetic features and signaling pathways in the used cell lines.

STAT3 also plays an important role in cell growth, survival, differentiation and gene expression via phosphorylation at Tyr705 followed by dimerization, translocation to the nucleus and DNA binding. STAT3 phosphorylation at Ser727 is associated with its role as a transcription factor [19]. Although the latter phosphorylation site was not affected, increased STAT3 phosphorylation at Tyr705 was observed after CRIP1 knockdown in T47D cells. This outcome indicates an association of CRIP1 with selective STAT3 activation, and reduced CRIP1 protein levels increase cell proliferation and survival via STAT3 activation *in vitro*.

We also determined the activation of Akt through phosphorylation at Thr308 and Ser473 using Western blot analysis. Activated Akt regulates survival and apoptosis through inhibiting target proteins [17,33]. After CRIP1 knockdown, we observed an increase in Akt phosphorylation at Thr308 that may cause reduction in anti-apoptotic signaling. These results indicate that CRIP1 is associated with Akt.

Because CRIP1 knockdown did not affect the phosphorylation of p38 MAPK or PTEN, we conclude that p38 MAPK- and PTEN-mediated signal transduction is independent of CRIP1 expression levels.

After CRIP1 knockdown, we also analyzed the *in vitro* phosphorylation status of cdc2, a cell cycle protein that is involved in the entrance into mitosis [34,35]. CRIP1 silencing led to a slight reduction of phosphorylation of cdc2 at Tyr15 and a consequential increase in the activation of this cell cycle protein, which again suggests that cell proliferation increases at low CRIP1 levels. In addition, our Western blot results were underpinned by significantly increased proliferation *in vitro* when CRIP1 was downregulated in T47D and BT474 breast cancer cells. Recently, Jeschke et al., also described CRIP1 as a potential prognosticator for poor overall survival in breast cancer based on the methylation of CRIP1 gene promoter which may lead to its silencing [36]. This fully agrees with our study demonstrating that downregulation of CRIP1 in breast cancer cell lines rather leads to increased cell proliferation and invasion and this may also result in a poor prognosis for breast cancer patients.

In this study, we aimed to further characterize CRIP1 in breast cancer. We identified CRIP1 as an independent prognostic factor of the metastases-free survival of breast cancer patients and found that, in HER2-positive tumors, CRIP1 expression allowed for the identification of two distinct prognostic groups, with a better prognosis for patients whose tumors exhibited CRIP1 and HER2 expression. These results show that CRIP1 may serve as an additional therapeutic and prognostic marker, particularly in HER2-positive tumors. Furthermore, the results of our *in vitro* analyses indicate a possible tumor suppressor role for CRIP1 because its silencing was favorable for tumor cell proliferation, tumorigenic signaling and the invasive potential of breast cancer cells.

## Conclusions

CRIP1 was shown to be associated with HER2 expression in breast cancer tumors, but its function is still unclear. We show that in invasive breast carcinomas, CRIP1 expression is associated with not only HER2 expression but also the metastases-free survival of patients, with a more favorable prognosis for patients with high CRIP1 expression. In HER2-positive tumors, two distinct prognostic groups could be identified according to their CRIP1 expression.

The downregulation of CRIP1 in T47D and BT474 breast cancer cells resulted in the activation of signal transduction molecules (MAPK and Akt) and cyclin-dependent kinase (cdc2) and caused an increase of cell proliferation and invasion *in vitro*.

Our results demonstrate that low CRIP1 expression promotes increased cellular proliferation and the invasion of cells *in vitro* and is associated with a worse prognosis for breast cancer patients. Therefore, CRIP1 represents an additional prognostic marker in breast cancer.

## Materials and Methods

### Tumor samples

Ethical approval concerning the use of tumor tissues in this study was obtained from the Ethics Committee of the Medizinische Fakultät der Technischen Universität, Munich, Germany. All experimental research described here was performed on human tissue only and was in compliance with the Helsinki Declaration. Formalin-fixed and paraffin-embedded archival material was randomly collected from 113 patients with invasive ductal breast carcinomas. In total, 67 of the tumors were node-negative, and most of the tumors (n = 72) were less than 2 cm in size. According to the histological grade [37], most of the tumors were classified as grade 2 (n = 75), 9 as grade 1, and 29 as grade 3. In addition to the standard histopathological parameters (lymph node status, tumor size, histological type and grade), immunohistochemical data from the tumors were available for HER2 and estrogen and progesterone receptor. The median follow-up of patients was 134 months (max. 468 months), with 49 (44%) of the patients showing disease recurrence with distant metastases within the period of clinical follow-up.

### Tissue microarrays

Tissue microarrays (TMAs) were produced as previously described [38] using a tissue-arraying instrument (Beecher Instruments Inc., Silver Spring, MD, USA). Hematoxylin- and eosin-stained sections of the TMAs were examined, and the original paraffin blocks were re-examined to validate representative sampling.

### Immunohistochemical analyses

Immunohistochemical staining was performed on 3 μm thick sections of the TMAs using an automated stainer (Discovery XT) and a DAB Map kit (both Ventana Medical Systems, Tucson, AZ, USA). The CRIP1 primary antibody (AbD Serotec, Oxford, UK) was diluted 1:100, and the staining intensities were scored by two independent observers using a 4-point scale as indicated: 0 (no staining) and from 1+ (light staining) to 3+ (strong staining).

### Statistics

The correlations between CRIP1, HER2, and the histopathological parameters were examined with Spearman's rank correlation test. For univariate survival analyses, Kaplan Meier curves were calculated, and the differences between strata were evaluated with the log-rank chi-squared test. A multivariate analysis was performed using Cox proportional hazards regression and a stepwise selection algorithm (SAS Institute, Cary, NC, USA)*.* All of the parameters showing a significance level of *p* ≤ 0.15 in univariate analysis were analyzed with multivariate analysis. In all of the other tests, statistical significance was established if *p* ≤ 0.05.

### Cell culture and transient silencing of CRIP1

The human T47D and MCF7 breast cancer cell lines were maintained in RPMI 1640 (Roswell Park Memorial Institute) medium. The human BT474, SKBR3, MDA-MB-231, MDA-MB-361 and JIMT breast cancer cell lines were maintained in DMEM (Dulbecco’s Modified Eagle Medium). The media were supplemented with 10% FBS, the antibiotics penicillin and streptomycin (0.5%), 10 μg/ml human insulin (for the T47D, MCF7 and JIMT-1 cells), and the cells were maintained at 37°C in 5% CO_2_. To identify efficient and specific siRNAs for the knockdown of CRIP1, T47D and BT474 breast cancer cells were transiently transfected with four different siRNAs (Invitrogen, Carlsbad, CA, USA, and Santa Cruz Biotechnology, Heidelberg, DE) and positive and negative control siRNAs for 48 h and 72 h, as described previously [39]. Specific transfections were performed in three independent experiments.

### Western blot analysis

For SDS-PAGE and Western blot analysis, T47D and BT474 breast cancer cells were treated as described previously [39]. The proteins were detected with primary antibodies targeting CRIP1 (AP4707b, Abgent, San Diego, CA, USA); HER2 (A0485, DAKO, Glostrup, DK); (phospho, 9554) PTEN (9559), (phospho, 4376) MAPK (4695), (phospho, 9211) p38 MAPK (9212), phospho-STAT3 (9131 and 9134), (phospho, 4056) Akt (9272), phospho-cdc2 (9111), and MMP9 (3852) (Cell Signaling Technology, Beverly, MA, USA); cdk2 (sc-6248) and GAPDH (sc-25778) (Santa Cruz Biotechnology, Heidelberg, DE); STAT3 (610190, BD Transduction Laboratories, Lexington, KY, USA); and actin (A5441) and tubulin (T5168, Sigma, St. Louis, MO, USA). Anti-rabbit (NA934) and anti-mouse (NA931) peroxidase-conjugated secondary antibodies were obtained from GE Healthcare (Chalfont St. Giles, Buckinghamshire, UK). All bands showing altered intensities after CRIP1 knockdown were quantified relative to the control bands using the Molecular Imager ChemiDoc™ XRS and the analysis software Quantity One® (Bio-Rad Laboratories, Hercules, CA, USA).

### WST-1 cell proliferation assay

Cell proliferation was determined using water-soluble tetrazolium WST-1 (4-[3-(4-Iodophenyl)-2-(4-nitrophenyl)-2H-5-tetrazolio]-1,3-benzene disulfonate) for the spectrophotometric assay according to the manufacturer’s protocol (05015944001, Roche Diagnostics, Mannheim, DE). One day after transfection, T47D and BT474 cells were seeded at a concentration of 1 × 10^4^ cells per well in a 96-well tissue culture plate. After following 48 h, the WST-1 reagent was added and the cells were incubated for 0.5 h to 4 h at 37°C. The absorbance of the infected and the control cells was measured against a background control using a microplate ELISA reader (Bio-Rad, München, DE) at 450 nm (reference wavelength at 655 nm). Five independent experiments were performed.

### Wound scratch migration assay

A migration assay using transiently transfected and nontransfected T47D breast cancer cells was performed twice and quantified as described previously [39]. In brief, a confluent monolayer of T47D cells was scratched using a 1 mm pipette tip. The cells were washed, and serum-reduced medium was added at a concentration (0.1% FBS) that reduced proliferation but was sufficient to avoid apoptosis or cell detachment [40]. The cells were incubated at 37°C in 5% CO_2_ and monitored.

### Matrigel invasion assay

Control and transfected T47D and BT474 breast cancer cells were seeded at a density of 5 × 10^4^ onto BD BioCoat Matrigel Invasion Chambers (BD, Bedford, MA, USA) in 24-well cell culture plates and incubated for 24 h (T47D cells) or 48 h (BT474 cells) at 37°C one day after transfection. For T47D cells, epidermal growth factor (EGF, 25 ng/ml in serum-reduced medium) was used as a chemoattractant in the lower chamber. For the BT474 cells in the lower chamber the complete medium was used and the invasion assays were performed according to the manufacturer’s instructions. After incubation, the non-invading cells were removed from the apical side of the membrane with a cotton swab. The invading cells were then fixed with methanol, stained with toluidine blue, and counted under a microscope. The assay was performed twice.

## Abbreviations

CRIP1: Cystein rich intestinal protein 1; cdc: Cell division control protein; cdk: Cyclin-dependent kinase; ER: Estrogen receptor; FBS: Fetal bovine serum; GAPDH: Glyceralaldehyde 3-phosphate dehydrogenase; HER: Human epidermal growth factor receptor; LIM: Lin-11, Isl-1, Mec-3; IDC: Invasive ductal carcinoma; MAPK: Mitogen-activated protein kinase; MMP: Matrix metalloproteinase; PI3K: PHOSPHATIDYLINOSITOL 3-kinase; PR: Progesterone receptor; PTEN: Phosphatase and tensine homologue deleted on chromosome ten; RNAi: RNA interference; siRNA: Small interfering RNA; STAT: Signal transducer and activator of transcription; TMA: Tissue micrarray.

## Competing interests

The authors declare that they have no competing interests.

## Authors’ contributions

NL and MA analyzed and interpreted the data and drafted the manuscript. MA and AW supervised the study, and carried out the evaluation of the immunohistochemical stainings and image analysis. SE, KP and SR participated in acquisition of data. GA signed responsible for histopathological examination of tumor samples and assembly of tissue microarrays. HB performed the statistical analyses and interpreted the data. HH was involved in conception and design and coordinated this study. All authors read and approved the final manuscript.

## Supplementary Material

Additional file 1 The original Western blots of CRIP1-deleted and mock T47D cells using antibodies targeting CRIP1, GAPDH and Tubulin. Click here for file
