# Supplementary figures and images for: The impact of Cysteine-Rich Intestinal Protein 1 (CRIP1) in human breast cancer
Source: Mol Cancer. 2013 Apr 9;12:28. doi: 10.1186/1476-4598-12-28 (PMC3666946; doi:10.1186/1476-4598-12-28)

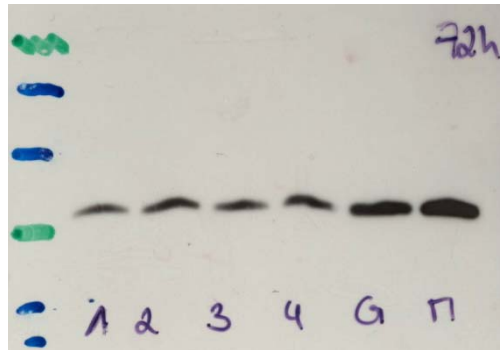

CRIP1

1: siRNA1  
 2: siRNA2  
 3: siRNA3  
 4: siRNA4  
 G: siRNA\_GAPDH  
 M: mock

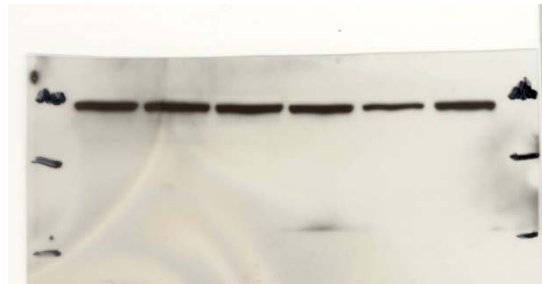

GAPDH

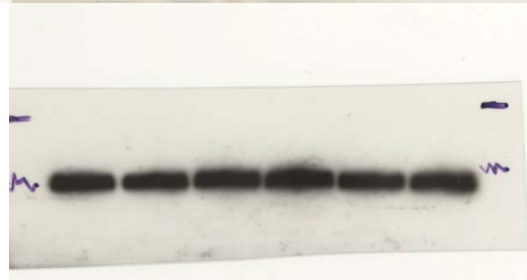

Tubulin

Supplement: Additional file 1 — The original Western blots of CRIP1-deleted and mock T47D cells using antibodies targeting CRIP1, GAPDH and Tubulin. [file 1476-4598-12-28-S1.pdf]
